# Microstructure and Mechanical Properties of Intergranular Boride Precipitation-Toughened HfMoNbTaTiZr Refractory High-Entropy Alloy

**DOI:** 10.3390/ma15196666

**Published:** 2022-09-26

**Authors:** Ping-Hsu Ko, Ya-Jing Lee, Shou-Yi Chang

**Affiliations:** 1 Department of Materials Science and Engineering, National Tsing Hua University, Hsinchu 30013, Taiwan; 2 High Entropy Materials Center, National Tsing Hua University, Hsinchu 30013, Taiwan

**Keywords:** refractory alloy, high-entropy alloy, boride, grain boundary, mechanical property

## Abstract

To develop strong refractory high-entropy alloys for use at elevated temperatures as well as to overcome grain-boundary brittleness, an equimolar HfMoNbTaTiZr alloy was prepared, and a minor amount of boron (0.1 at.%) was added into the alloy. The microstructures of the alloys were characterized, and their macro-to-microscale mechanical properties were measured. The microstructural observations indicated that the matrices of both the alloys were composed of a body-centered cubic solid-solution structure, and the added boron induced the precipitation of hexagonal close-packed borides (most likely the (Hf, Zr)B_2_) at the grain boundaries. The modulus and hardness of differently oriented grains were about equivalent, suggesting a diminished anisotropy, and many small slips occurred on multiple {110} planes. While the hardness of the matrix was not increased, the intergranular precipitation of the borides markedly raised the hardness of the grain boundaries. Owing to the enhanced grain boundary cohesion, the work hardenability and ductility were effectively improved with the addition of boron.

## 1. Introduction

With the needs of robust materials for use in elevated-temperature environments, high-temperature materials have been strongly demanded in recently years. However, the insufficient strength of nickel-based superalloys at temperatures higher than 1200 °C limits the further development of more efficient turbine-jet engines in the aerospace industry [1]. The design concept of high-entropy alloys (HEAs) with equimolar or near-equimolar ratios of multiprincipal elements has been proposed for about two decades [2]. Since that, many new stable solid-solution alloy systems without the formation of detrimental brittle phases have been developed [2,3,4,5]. Among them, body-centered cubic (BCC) refractory high-entropy alloys (RHEAs) are of great interest as they show very good mechanical performance such as high retaining strengths or lessened softening at elevated temperatures owing to their special dislocation activities [6,7,8]. The first developed single-phase WTaMoNbV RHEA even had a yield strength of about 400 MPa at the high temperature of 1600 °C, but the high density and low room-temperature ductility render its industrial application limited [9,10]. Subsequently, the HfNbTaTiZr solid-solution RHEA with a lower density and a higher ductility was developed, but its relatively low strength and particularly the serious high-temperature softening countervail the advantage of the high elevated-temperature strength of typical RHEAs [11,12,13]. For single-phase solid-solution RHEAs, although the mechanical properties may be improved by the adjustment of the compositions, the strength–ductility trade-off problem remains [14], and the development of other robust RHEAs is still demanded.

In recent years, heterostructural HEAs with microstructural heterogeneity to generate plastic discontinuity and thus mechanical strengthening have been intensively developed for overcoming the strength–ductility trade-off problem [15,16,17,18]. The proposed heterostructures include multiphase (most often called complex concentrated alloys), eutectic dual-phase and single-phase (grain-size gradient) structures [19,20,21]. Among them, the precipitation-strengthened heterostructure without the need of marked changes in the fabrication process is more efficient and can be applied to the industry more easily. By adjusting compositions or adding minor alloying elements to form fine precipitates in the matrix or at the grain boundaries, local stress accumulations are thereby expected to activate special deformation mechanisms and alter the dislocation gliding modes for significantly strengthening the alloys [22]. The strengthening effect depends on the coherency between the precipitates and the matrix; e.g., the yield strength and elongation of the (FeCoNi)_86_-Al_7_Ti_7_ alloy with the precipitation of L1_2_ phase even reach 1 GPa and 50%, respectively [23,24,25]. Boron has also been widely added in alloys to improve the cohesive strength of grain boundaries [26], e.g., to change the fracture mode of Ni_3_Al from intergranular to transgranular and thus prevent early failure [27], or to lower the migration rate of grain boundaries and inhibit grain growth at high temperatures [28]. Additionally, the short-range order in an Fe_40_Mn_40_Co_10_Cr_10_ alloy caused by the addition of boron atoms or the precipitation of borides was reported to retard the motion and increase the density of dislocations [29], and the discontinuous intergranular precipitation of borides in high-strength steels avoided the intergranular fracture [30], both improving the mechanical properties of the alloys [31,32]. As the grain-boundary brittleness is a major issue for BCC RHEAs, a minor amount of boron element (0.1 at.%) was hence added into an HfMoNbTaTiZr RHEA in the present study for potential precipitation of borides at the grain boundaries and improvement of grain boundary cohesion, then to yield a larger work hardenability and better ductility.

## 2. Materials and Methods

Equimolar HfMoNbTaTiZr RHEA samples (denoted as RHEA) were prepared by arc melting the constituent elements Hf, Mo, Nb, Ta, Ti and Zr (high purity > 99.99%) in vacuum (about 1.3 Pa) and the casting of ingots in a water-cooled Cu mold. Before that, the chamber was purged with pure argon and vacuumed, and Ti was melted to absorb the remaining oxygen in the chamber. For the preparation of HfMoNbTaTiZr RHEA samples with 0.1 at.% boron (denoted as RHEA-B), a proper minor amount of TiB_2_ was added. For a high chemical homogeneity, each sample was remelted three times at least. The cast samples were then homogenized at 1200 °C for 24 h and water quenched. The crystal structures of RHEA and RHEA-B samples were determined by using X-ray diffraction (XRD, D2 PHASER, Bruker, Billerica, MA, US) from 20° to 80° at a scanning speed of 6°/min, and the lattice parameters were calculated from the diffraction peaks. The microstructures were observed by applying a field-emission scanning electron microscope (SEM, SU-8010, Hitachi, Japan) equipped with energy dispersive spectrometry (EDS, Horiba, Japan), and the chemical compositions were determined by using SEM EDS and the wavelength dispersive spectrometry (WDS) of electron probe microanalysis (EPMA, JXA-8500F, JEOL, Japan). The crystal orientations and composing phases were identified by applying a field-emission SEM (Supra 55, Zeiss, Germany) equipped with an electron backscatter diffraction (EBSD, AztechHKL, Oxford, UK).

The hardness of the bulk RHEA and RHEA-B samples were measured by using a Vickers hardness tester, while the micro-to-nanoscale elastic modulus and hardness of the differently oriented single grains (〈100〉, 〈110〉 and 〈111〉, as identified by EBSD) were measured by applying instrumented nanoindentations (Hysitron Triboindenter® TI 980, Bruker, Billerica, MA, US) with the XPM (accelerated property mapping) mode. The contact area of the Berkovich indenter (diamond tip radius of 100 nm) was calibrated by using fused silica, and the load was ramped to 8000 μN for the indentation tests. The deformation behavior of the alloy samples was characterized by conducting in situ micropillar compression tests in an SEM. Single-crystalline 〈100〉 oriented micropillars were first cut (with a top Pt protective coating at the cutting locations) from a corresponding grain by using a focused ion beam system (FIB, NX 2000, Hitachi, Japan) and were milled to a diameter of about 2 μm and a length of 5 to 6 μm with an ultralow current of below 50 pA for preventing the ion damage of the surface. The micropillars were then in situ compressed in an SEM (JSM-IT500, JEOL, Japan) by using a microcompression module (Hysitron Picoindenter® PI 88, Bruker, Billerica, MA, USA) in a displacement-controlled mode at a strain rate of 10^−^^3^/s for a total strain of 20%. For macro-compression tests, cylindrical specimens of bulk polycrystalline RHEA and RHEA-B samples were prepared by electric discharge cutting to a diameter of 5 mm and a height of 8 mm and were ground and polished. The compression tests were then conducted by applying the Instron 4468 tester at a strain rate of 10^−^^3^/s to the fracture of the specimens, and the fractographies were observed by using an SEM (JEOL JSM-IT500).

## 3. Results and Discussion

### 3.1. Basic Properties and Crystal Structure

The design rule of equimolar HfMoNbTaTiZr RHEA was based on the properties of previous WTaMoNbV and HfNbTaTiZr RHEAs: W was removed due to the high density and the low ductility of WTaMoNbV, and Mo was added owing to the low elevated-temperature strength of HfNbTaTiZr. Minor 0.1 at.% B boron was added for precipitation strengthening and improving the property of grain boundaries. Table 1 provides the basic properties of the constituent metallic elements in the RHEA [33,34,35,36,37,38,39,40], and Table 2 lists the mixing enthalpies between each two elements [41]. According to the calculation rule of lattice distortion [42], the parameter delta δ of the RHEA sample is expected to be as high as about 5.8%. Several advantages of the RHEA are anticipated: (1) the much larger atomic sizes of Hf and Zr than others to generate a large lattice distortion and enhance the solid-solution strengthening, (2) the high modulus of Mo to raise the stiffness, (3) the high melting points of Nb and Ta (BCC stabilizers) to elevate the strength at elevated temperatures and (4) the light-weight Ti to lower the density of the alloy.

**Figure 1:**
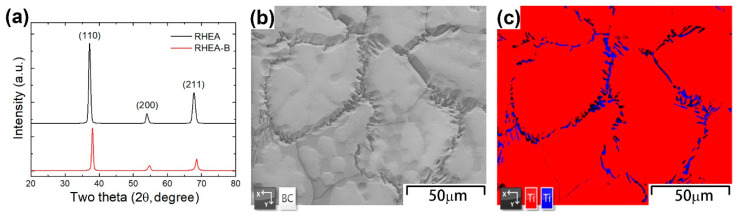
(**a**) XRD patterns of RHEA and RHEA-B samples; (**b**) SEM image and (**c**) EBSD phase map of RHEA-B sample (red: BCC; blue: HCP; standard: the phases of Ti element).

From the small mixing enthalpies between each of the two elements given above and the great contribution of the high mixing entropy, the formation of a simple solid-solution structure without intermetallic compounds is expected, as also identified by the XRD and EBSD analyses in Figure 1. The XRD patterns in Figure 1a indicate the formation of a single-phase BCC structure in both the RHEA and RHEA-B samples. Owing to the higher cohesive energy of boron to the metallic elements than that of the metallic-to-metallic constituents [26], a smaller lattice parameter was expected, and thus a slight shift of the diffraction peaks to a higher angle was observed upon the addition of boron. However, as more carefully characterized in the SEM image and EBSD phase map in Figure 1b,c, besides the major BCC phase composing the matrix of the RHEA-B sample, the minor addition of boron segregating at the grain boundaries was observed to induce the intergranular precipitation of the second phase (hexagonal close-packed, HCP) along the grain boundaries although the amount of the second phase was too small to be efficiently identified in the XRD analyses.

### 3.2. Microstructure and Chemical Composition

**Figure 2:**
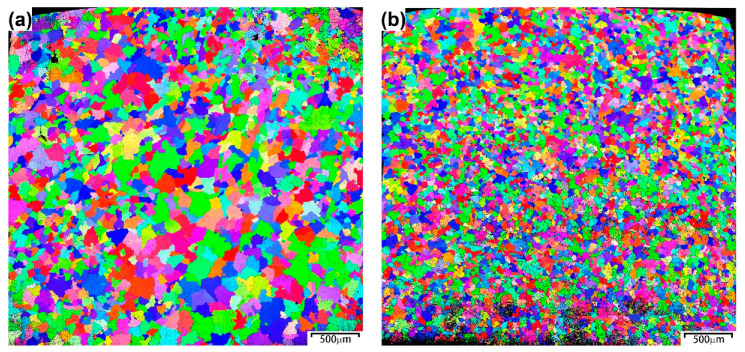
EBSD IPF maps of (**a**) RHEA and (**b**) RHEA-B samples showing the microstructure and different grain orientations.

The EBSD IPF maps in Figure 2 show the microstructure of the RHEA and RHEA-B samples and indicate the random orientations of the equiaxed grains in the homogenized samples without a texture or a preferred orientation. The grain size of the RHEA was about 130 μm, but the grain size of the RHEA-B was much smaller, only about 40 μm. As suggested above in Figure 1b, the addition of boron and the intergranular precipitation of HCP borides were believed to hinder the migration of grain boundaries and thus limit the growth of grains during homogenization at 1200 °C, leading to an effective grain refinement.

**Figure 3:**
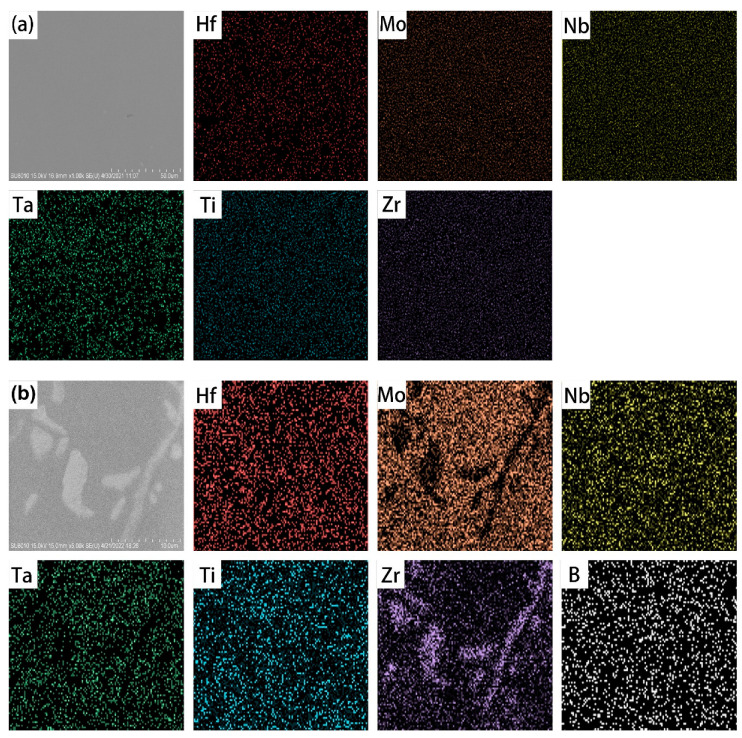
SEM EDS elemental mappings showing (**a**) the uniform distribution of constituent elements in RHEA sample but (**b**) the segregation of Hf and Zr in RHEA-B sample.

Figure 3 show the SEM EDS elemental mappings of the RHEA and RHEA-B samples, and Table 3 lists the chemical compositions of the samples in different regions. Basically, the average data were close to the designed compositions, and the constituent metallic elements were uniformly distributed in the RHEA sample and the matrix of the RHEA-B sample. As mentioned above, the segregation of Hf and particularly Zr to generate borides at the grain boundaries of the RHEA-B sample, leaving slightly higher contents of Mo, Nb, Ta and Ti in the matrix than the average level, was obvious. However, due to the low atomic weight of boron and the detection limit of SEM EDS analysis, the EDS was not sufficient to accurately identify the amount of boron in the precipitates. The chemical analysis was thus additionally carried out by using EPMA WDS, which indicated that the enrichment of boron in the region of grain boundaries with the precipitates was about 3–5 at.%, which is low (due to the low atomic weight of boron and the large detection area); however, it was much higher than the minor-added amount, 0.1 at.%. According to the mutual solubility of Hf and Zr as well as their very large negative mixing enthalpies with boron (the formation heats of borides: HfB_2_ −90 kJ/mole and ZrB_2_ −98 kJ/mole [42]), precipitates in the form of (Hf, Zr)B_2_ at the grain boundaries of the RHEA-B sample are expected, which is consistent with the observed HCP phase and the high diffusivity of boron atoms to the grain boundaries [43].

### 3.3. Nanoindenting Modulus and Hardness

**Figure 4:**
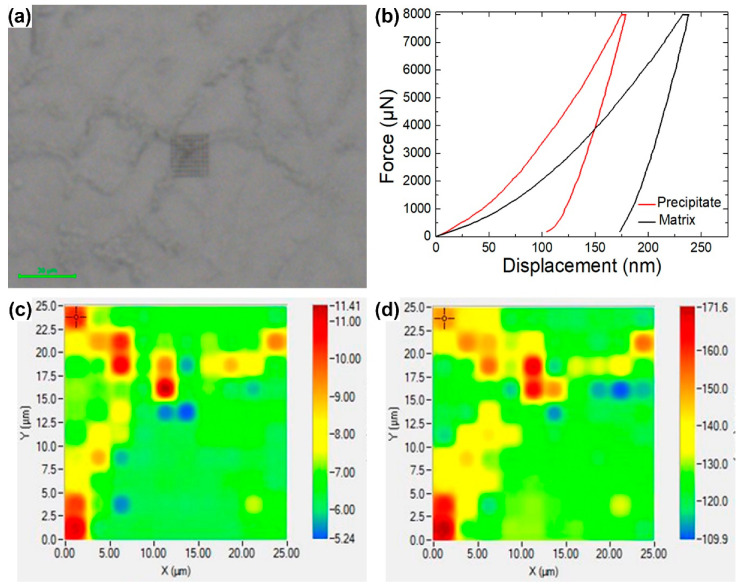
Nanoindentation mappings of RHEA-B sample: (**a**) mapping area (central dark grey square), (**b**) representative load–displacement curves in the matrix (grain) and at the precipitate (grain boundary), (**c**) reduced modulus mapping, (**d**) hardness mappings.

**Figure 5:**
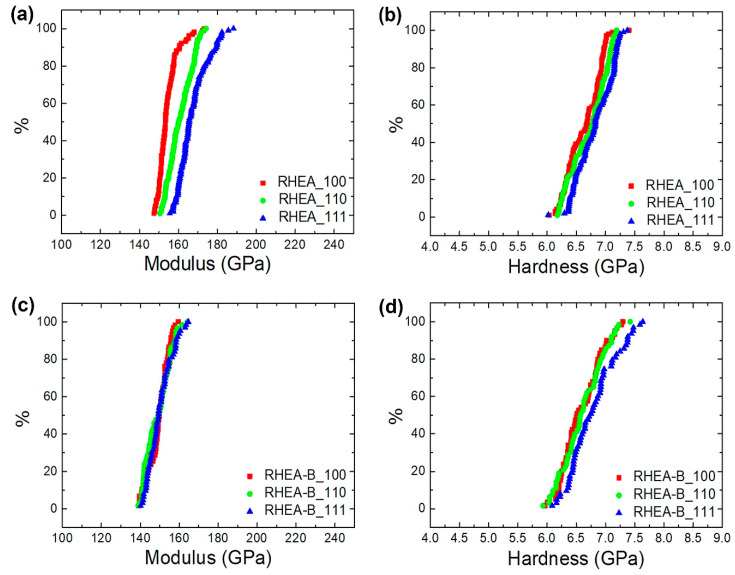
Accumulative plots of nanoindentation modulus and hardness for the 〈100〉, 〈110〉 and 〈111〉 grains of RHEA and RHEA-B samples: (**a**) modulus of RHEA, (**b**) hardness of RHEA, (**c**) modulus of RHEA-B, (**d**) hardness of RHEA-B.

The Vickers hardness of the bulk RHEA and RHEA-B samples were measured to be HV 473 (±7.0) and HV 477 (±1.0), respectively, which were lower than that of WTaMoNbV, HV 535 [8,10], but much higher than that of HfNbTaTiZr, HV 390 [11]. The addition of boron was not observed to raise the hardness of the bulks as the amount of solutes was only 0.1 at.% and many of them segregated to the grain boundaries, as also verified by the nanoindentation tests. Figure 4 further presents the nanoindentation mappings of the RHEA-B sample around a grain junction area, and Figure 5 shows the accumulative plots of the nanoindentation modulus and hardness of the RHEA and RHEA-B samples for differently oriented grains (〈100〉, 〈110〉 and 〈111〉). The results indicated that, consistent with the Vickers hardness tests, the mechanical properties of the matrices of both the samples were almost equivalent. More importantly, the modulus and hardness of the precipitates (the grain boundaries; modulus of 160 to 170 GPa and a hardness of 10 to 11 GPa) were markedly higher than those of the matrix (the grains; modulus of 120 to 130 GPa and a hardness of 6.5 to 7.0 GPa).

Additionally, as revealed in Figure 5, a diminished mechanical anisotropy in either the RHEA sample or the RHEA-B sample is another interesting behavior of HEAs [44]. While the modulus and hardness of the 〈111〉 grains were slightly higher than those of the 〈110〉 grains and particularly the 〈100〉 grains (a higher hardness owing to a smaller Schmid factor along the 〈111〉 direction), the mechanical properties of the differently oriented grains were very close. Attributable to the disordered distribution of constituent atoms and the consequent random chemical bonds [44] and local chemical fluctuations [45], the neighboring bindings and the energy barriers for activating a dislocation motion were altered, therefore causing a decrease in anisotropy in both the elastic and plastic perspectives.

### 3.4. Micropillar Compression and Deformation

**Figure 6:**
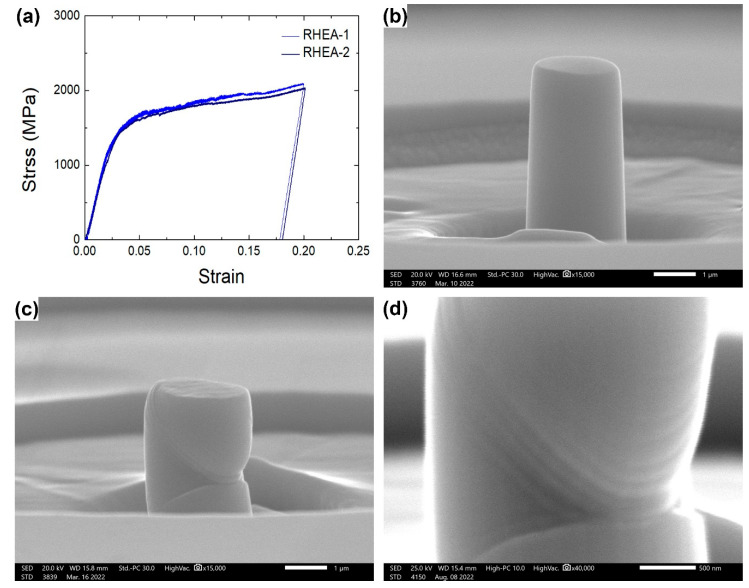
In situ SEM compression tests of 〈100〉 oriented single-crystalline micropillars of RHEA sample: (**a**) stress–strain curves, (**b**) SEM image of micropillar before compression, (**c**,**d**) SEM images of compressed micropillars.

Figure 6 shows the in situ SEM compression tests of the 〈100〉 oriented single-crystalline micropillars of the RHEA sample. From the stress–strain curves, the 0.2% yield stress was determined to be 1510 MPa, and the compressive stress at a strain of 20% was 2060 MPa. The SEM images of the compressed micropillars present typical slip deformation, with an inclined angle of 45° about the stress axis 〈100〉, suggesting the activation of dislocation gliding on the regular {110} 〈111〉 slip systems in BCC alloys with a large Schmid factor of 0.408. However, different from the drastic plastic deformation of traditional BCC alloys [46], the stress drops were relatively small, corresponding to the observed multiple small slip lines. It is expected that, owing to the large lattice distortions caused by the incorporation of multiprincipal elements with different atomic sizes, the defect activities would change from the long-distance gliding of few, long perfect dislocations to the short-distance gliding of a high density of fragmented partial dislocations (or stacking faults) on multiple {110} slip planes at the same time, therefore leading to the small altitudes of many stress drops.

### 3.5. Macro-Compressive Strength and Ductility

**Figure 7:**
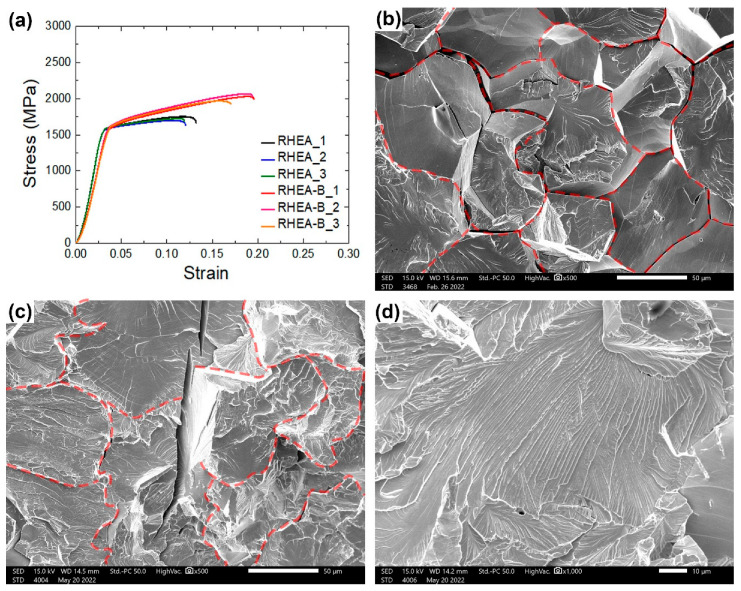
Macro-compression tests of RHEA and RHEA-B samples: (**a**) stress–strain curves, (**b**) SEM fractography of RHEA, (**c**,**d**) SEM fractographics of RHEA-B (red dashed lines: grain boundaries).

The minor addition of boron effectively improved the mechanical properties of the alloy, as presented in the macro-compressive stress–strain curves and fractographies of the RHEA and RHEA-B samples in Figure 7, and as the yield stresses, ultimate strengths, fracture strains and work hardening percentages listed in Table 4. While the yield stress only slightly increased from 1.57 GPa for the RHEA (close to the value obtained by the above micropillar compression, 1.51 GPa) to 1.61 GPa for the RHEA-B, the work hardenability and ductility were more obviously improved. The ultimate compressive strength of the RHEA, 1.75 GPa at a fracture stain of 12.3%, showed a similarly low hardenability (for only about 11%) and ductility to typical refractory alloys. In comparison, The RHEA-B exhibited a much higher ultimate compressive strength of 2.05 GPa (hardening for 27%) and fracture stain up to 19.3%. As observed in the fracture surfaces, attributable to the precipitation of borides at the grain boundaries much enhancing the boundary cohesion, the failure mode changed from a mainly intergranular facture for the RHEA sample (as the grain-boundary cracking as marked) to a major portion of the transgranular fracture for the RHEA-B sample (almost no fracture at the grain boundaries as marked). The minimized grain boundary decohesion inhibited the early failure of the RHEA-B, thus effectively improving the mechanical properties.

## 4. Conclusions

An equimolar HfMoNbTaTiZr RHEA was developed in this study, and the addition of a minor amount of boron (0.1 at.%, RHEA-B) further overcame the grain-boundary brittleness. As indicated by the microstructural observations and chemical analyses, a simple BCC-phase, solid-solution structure was formed in the matrices of both the alloys. The addition of boron did not obviously increase the hardness of the matrix but induced the intergranular precipitation of the HCP-phase (Hf, Zr)B_2_ borides which markedly raised the hardness of the grain boundaries. Attributable to the lattice distortions caused by the random distribution of different constituent elements, a diminished anisotropy in the mechanical properties was noticed, and the deformation behavior was mediated by many small slips on multiple {110} planes. Owing to the enhanced grain boundary cohesion with the addition of boron and the intergranular precipitation of borides, the work hardenability and ductility of the alloy were effectively improved, and the failure mode obviously changed from a mainly intergranular facture to a major portion of the transgranular fracture.

## Figures and Tables

**Table 1:** Basic Properties of The Constituent Metallic Elements of RHEA [33,34,35,36,37,38,39,40] and The Average Properties of RHEA by The Rule of Mixture.

	Hf	Mo	Nb	Ta	Ti	Zr	RHEA
Density (g/cm^3^)	13.31	10.28	8.57	16.65	4.51	6.51	9.97
Modulus (GPa)	78	329	105	186	116	68	147
Atomic radius (Å)	1.55	1.45	1.45	1.45	1.40	1.55	1.48
Lattice constant (Å)	3.19	3.14	3.30	3.30	2.95	3.23	3.41

**Table 2:** Mixing Enthalpies Between The Constituent Metallic Elements of RHEA [41].

Mixing enthalpy (kJ/mole)	Hf	Mo	Nb	Ta	Ti	Zr
Hf	0	−4	4	3	0	0
Mo		0	−6	−5	−4	6
Nb			0	0	2	4
Ta				0	1	3
Ti					0	0
Zr						0

**Table 3:** Chemical Compositions of RHEA and RHEA-B Samples Measured by SEM EDS.

	Hf	Mo	Nb	Ta	Ti	Zr
RHEA (average)	16.89 ± 0.84	17.01 ± 0.85	16.72 ± 0.84	17.18 ± 0.86	15.79 ± 0.79	16.41 ± 0.82
RHEA-B (average)	17.90 ± 0.90	15.91 ± 0.80	17.01 ± 0.85	14.99 ± 0.75	14.75 ± 0.74	19.44 ± 0.97
RHEA-B (matrix)	16.10 ± 1.29	18.20 ± 1.18	18.35 ± 0.66	17.57 ± 1.02	15.73 ± 0.38	14.05 ± 1.62
RHEA-B (precipitate)	31.29 ± 0.71	1.60 ± 0.74	5.01 ± 0.52	2.34 ± 0.35	8.40 ± 0.55	51.36 ± 0.86

**Table 4:** Macro-Compressive Yield Stresses, Ultimate Strengths, Fracture Stains and Work Hardening Percentages of RHEA and RHEA-B samples.

	**Yield Stress (GPa)**	**Ultimate Strength (GPa)**	**Fracture Stain (%)**	**Work Hardening (%)**
RHEA	1.57 ± 0.01	1.75 ± 0.02	12.3 ± 0.5	11.5 ± 0.01
RHEA-B	1.61 ± 0.02	2.05 ± 0.04	19.3 ± 1.2	27.3 ± 0.03

## Data Availability

The data presented in this study are available on request from the corresponding author.
